# Influence of a simple cyst on kidney function

**DOI:** 10.3389/fmed.2024.1381942

**Published:** 2024-08-16

**Authors:** Vigen Malkhasyan, Tukhtasin Makhmudov, Yunus Gilfanov, Igor Semenyakin, Sergey Sukhikh, Bagrat Grigoryan, Dmitry Pushkar

**Affiliations:** ^1^Urology Department of the Russian University of Medicine, Moscow, Russia; ^2^Botkin’s Hospital Moscow Urological Center, Moscow, Russia; ^3^Clinical Hospital No. 1 Group of Companies Medsi Moscow, Moscow, Russia

**Keywords:** simple kidney cyst, renal cyst, kidney function, total kidney volume, single kidney volume, split glomerular filtration rate, renal parenchyma loss

## Abstract

**Introduction:**

The aim of the study was to estimate the influence of a simple kidney cyst on kidney function and to determine indications for surgical treatment.

**Materials and methods:**

In this prospective cohort study, we analyze data on 109 patients who sought counseling with a simple kidney cyst. Patients with solitary cyst of the right or left kidney, grade I-IIF according to the Bosniak classification, were included. Split glomerular filtration rate (sGFR) was calculated. The maximum size of the cyst, single kidney volumes (SKV) and the volume of the lost (atrophied) parenchyma were estimated with computed tomography (CT) scan of the urinary tract with contrast.

**Results:**

The average difference between the sGFR of a healthy and affected kidney cyst was 11 [8.70; 13.44] ml/min, which is a statistically significant value (*p* = 0.001). Correlation analysis revealed a statistically significant relationship between the proportion of lost parenchyma and the maximum cyst size: *p* = 0.37 with 95% CI [0.20; 0.52] (*p* = 0). A multivariate logistic regression model showed that a statistically significant factor influencing the likelihood of a significant decrease in sGFR is the percentage of lost kidney parenchyma (OR = 1.13; *p* = 0).

**Conclusion:**

The growth of kidney cyst causes atrophy of the renal parenchyma and a decrease in the sGFR of the affected kidney. An increase in the volume of the atrophied parenchyma leads to a decrease in the sGFR of the affected kidney.

## Introduction

Kidney cysts are a common disease that is more prevalent in the elderly (1). The median prevalence of simple kidney cysts is 7–10% depending on the study population and imaging modalities (2, 3). Most often, the disease is asymptomatic, and lumbar pain, hematuria, and symptoms associated with obstruction of the upper urinary tract occur in only 4% of patients with kidney cysts (4). The average size of a kidney cyst in most patients at the time of detection does not exceed 10 mm; with dynamic observation, further growth of the cystic formation is possible at an average rate of 1.6 mm or 5% of the initial cyst size per year. The size of cysts commonly doubles within 10 years, after which the growth of the cyst stabilizes (2, 5, 6). The malignant potential of simple kidney cysts (Bosniak I-II) is negligible and does not exceed 1% (7–9). For this reason, there are detailed, and clear clinical guidelines developed by many professional communities for the management of patients with complex renal cysts. However, there are still no clinical recommendations regarding the tactics of managing patients with simple kidney cysts. The absence of clearly defined criteria for surgically treating simple kidney cysts may contribute to this situation. The primary symptoms that frequently prompt individuals to seek medical attention and lead to the consideration of surgical intervention are typically pain and discomfort localized to the affected side. This underscores the importance of establishing standardized guidelines for determining the appropriate course of action in managing simple kidney cysts (10). However, there is evidence that a simple kidney cyst can have a negative impact on kidney function (11–20). The decrease in kidney function most likely occurs due to partial atrophy of the renal parenchyma (in the zone of the “crater” of the cyst) caused by compression. The aim of our study was to assess the impact of a simple kidney cyst on kidney function and to identify the characteristics of the cyst that affect kidney function, in order to determine indications for surgical treatment.

## Materials and methods

A prospective cohort study on the influence of a simple kidney cyst on kidney function was conducted. Patients who sought consultative care at the S.I. Spasokukotsky City Clinical Hospital in Moscow, Russian Federation, with kidney cysts and patients who had a simple kidney cyst during a screening examination or examination for another disease between February 16, 2022, and December 16, 2022, were included in the study. The inclusion criteria were as follows: adult patients with simple cyst of the kidney Bosniak I-II, solitary cyst of the kidney and unilateral lesion. The exclusion criteria were: inability to perform contrast-enhanced CT, other kidney cysts, cysts with a maximum size of less than 2 cm, a solitary kidney, glomerulonephritis, chronic pyelonephritis, amyloidosis, diabetes mellitus, autoimmune diseases, urolithiasis, kidney dystopia, non-functioning kidney, kidney hypoplasia, accessory kidney, operations on the kidneys and upper urinary tract, anomalies in the development of the kidneys and upper urinary tract with impaired urine outflow, calcification of the renal arteries, stenosis of the renal arteries, hypertensive nephropathy, severe concomitant diseases, chemotherapy, long-term (more than 10 days) antibiotic therapy in the last month, long-term (more than 7 days) use of NSAIDs for the last month, diagnostic and therapeutic manipulations accompanied by the introduction of X-ray contrast agents for 30 days. All patients signed informed consent to participate in this study. The study was approved by local Ethical Committee of the University of Medical Sciences on January 21, 2022 Patients underwent the following types of examinations: collection of anamnesis and complaints related to the presence of the cysts, physical examination, weighing and measurement of the height, CT scan of the urinary tract with contrast to determine the maximum size of the cyst, the single kidney volumes (SKV) and the volume of the lost (atrophied) parenchyma with an assessment of compression and deformation of the cyst of the pelvicalyceal system (PCS), Tc-99 m diethylenetriaminepentaacetic acid (DTPA) renal dynamic imaging with the calculation of total glomerular filtration rate (GFR) and split glomerular filtration rate (sGFR).

**Figure 1:**
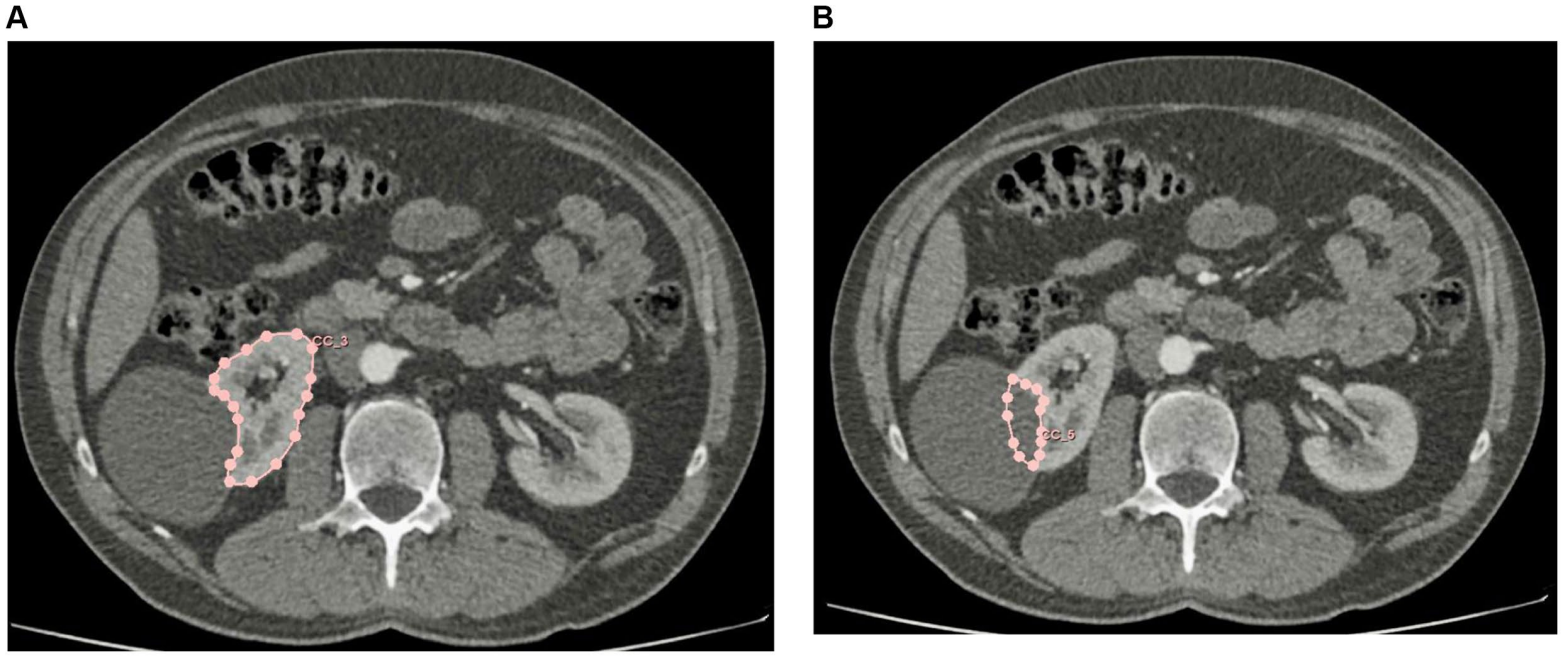
**(А)** Calculation of the volume of the renal parenchyma by segmentation. **(B)** Calculation of the volume of the cyst crater by segmentation.

The volume of the crater of the kidney cyst was considered as the volume of the atrophied parenchyma of the kidney. The actual volume of the renal parenchyma was calculated using the segmentation method (Figure 1). First, the area of the renal parenchyma was determined by contouring the latter on the axial section, after which the section volume was automatically calculated by the program by multiplying the area of the parenchyma by the length of the section. The volume of the renal parenchyma was calculated by summing the volumes of all segments (sections). The volume of the crater was also calculated using the segmentation method. When contouring the crater on the axial section, the outer contour of the crater was shaped by the researcher by drawing a curved line continuing the outer contour of the kidney (Figures 1A,B). The initial volume of the renal parenchyma was calculated by summing the actual volume of the renal parenchyma and the volume of the “crater” of the kidney cyst.

The symmetry of the function of both kidneys was analyzed by comparing the sGFR of the affected and healthy kidney, the relationship between the presence of a simple kidney cyst and a decrease in renal function compared to a healthy kidney, as well as the relationship between the maximum size of a kidney cyst and a decrease in renal function compared to the healthy kidney. The relationship between the volume of the atrophied parenchyma of the kidney and the decrease in its function compared to the healthy kidney was also analyzed.

## Statistical analysis

When analyzing quantitative data, preliminary testing of variables for the normality of distribution was carried out using the Shapiro–Wilk test. In the case of a normal distribution, the parameter was presented as an arithmetic mean and standard deviation (SD) (mean ± SD). In case of deviation from the normal distribution, the parameter was presented as a median and 1st and 3rd quartiles (Q1, Q3) [median (interquartile range, IQR)]. If necessary, 95 percent confidence intervals (CI) were built for the estimated parameters, which are given as [lower limit of CI; upper limit of CI].

When comparing parameters in the case of a normal distribution, a paired Student’s t-test was used, in the case of a logarithmic normal distribution, the t-test is applied to the logarithmic transformation of the original parameter. For other distributions, the Wilcoxon W-test for connected samples was used. A generalized linear model (GLM) was used to analyze the parameters influencing the sGFR of the affected kidney. To analyze the probability of a significant decrease in the sGFR of the affected kidney, as well as for the factors influencing this probability, a multivariate logistic regression model was built. The receiver operating characteristics (ROC) analysis was used to assess the quality of the model.

## Results

Data on 109 patients were available for analysis, of which 62 (56.9%) patients were male and 47 (43.1%) were female. The mean age of the patients was 62 (54; 68), mean body mass index (BMI, kg/m^2^) was 28.16 ± 4.07 kg/m^2^, mean creatinine level was 87.4 ± 20.93 μmoL/L. In 55 (50.5%) patients, the kidney cyst was located in the left kidney, and in 54 (49.5%) – in the right kidney. In 43 (39.5%) patients, the cyst was localized in the upper segment of the kidney, in 42 (38.5%) patients in the middle segment, and in 24 (22%) patients in the lower segment of the kidney. In 53 patients (48.6%), the deformation and compression of the pelvicalyceal system (PCS) of the kidney by the cyst were found (Table 1).

**Table 1:** Baseline characteristics of patients (*n* = 109).

Male, *n* (%)	62 (56.9%)
Female, *n* (%)	47 (43.1%)
Age, years	62 ± 10.7
Height, cm	169.6 ± 8.6
Weight, kg	81.5 ± 15.7
BMI, kg/m^2^	28.16 ± 4.07
Creatinine level, μmol/l	87.4 ± 20.93
Left kidney cyst, *n* (%)	55 (50.5%)
Right kidney cyst, *n* (%)	54 (49.5%)
Upper kidney segment cyst, *n* (%)	43 (39.5%)
Middle kidney segment cyst, *n* (%)	42 (38.5%)
Lower kidney segment cyst, *n* (%)	24 (22%)
Compression of the pelvicalyceal system (PCS), *n* (%)	53 (48.6%)

The median of the maximum cyst size was 80 (66; 97) mm. The largest maximum size of the cyst was 201 mm, the minimum was 46 mm. The median volume of the parenchyma of the affected kidney was 174 (IQR 137–206) ml, the median volume of the atrophied or lost parenchyma (the volume of the cyst crater) was 49 (IQR 27–71) ml. The median proportion of lost parenchyma was 28% (IQR 19–37). The median total GFR was 77.07 (IQR 66.8–90.8) ml/min.

**Figure 2:**
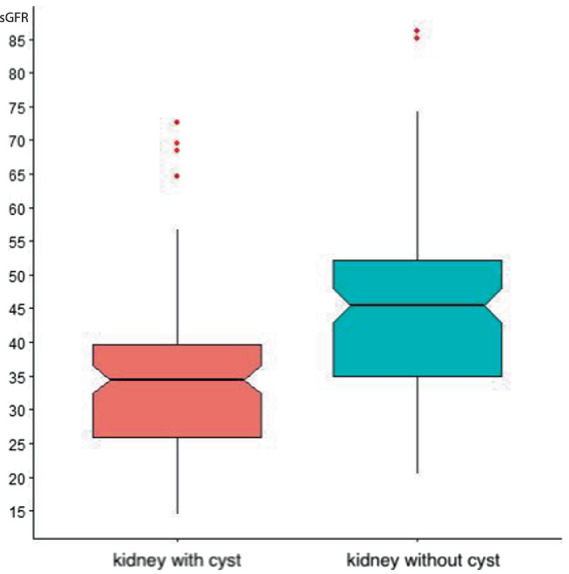
Comparison of sGFR in a healthy and diseased kidney.

72 x serum creatinine (in mg/dL). The median sGFR in a healthy kidney was 45.49 (IQR 35.03–52.07) ml/min, and the median sGFR R in an affected kidney cyst was 34.46 (IQR 25.97–39.63) ml/min. The average difference between the sGFR of a healthy and affected kidney cyst was 11.00 [8.70; 13.44] ml/min, which is statistically significant (*p*-value = 0) (Figure 2).

Correlation analysis showed that the proportion of lost parenchyma was statistically significantly correlated with the maximum cyst size: ρ = 0.37 with 95% CI [0.20; 0.52] (*p*-value = 0). Therefore, only parenchyma loss was considered in further models, and the maximum cyst size was excluded from the models.

As a generalized linear model for the logarithm of the sGFR of the affected kidney, a model was chosen with the following parameters: gender, age, BMI, the logarithm of the proportion of lost parenchyma, and the presence of compression or deformation of the PCL of the kidney by a cyst. Statistical analysis showed that a significant (at a significance level of 5%) parameter that negatively affects the change in sGFR of a kidney with a cyst is the proportion of lost parenchyma (Table 2).

**Table 2:** Generalized linear model of the logarithm of the sGFR in a kidney with a cyst.

Parameter	Coefficient estimation	Standard error	*p*-value
Intercept	1.416	0.409	0.001
Sex (m)	−0.007	0.042	0.876
Age	−0.003	0.002	0.192
BMI	0.003	0.005	0.515
Percentage of lost parenchyma	−0.01	0.002	0
The presence of compression of the PCS	0.005	0.042	0.906

To assess the probability of a significant decrease in sGFR (by more than 10 mL/min) and to identify factors that statistically significantly affect this probability, a multivariate logistic regression model was constructed. According to the results of the analysis, a statistically significant factor influencing the probability of a significant decrease in sGFR is the proportion of lost kidney parenchyma, an increase in the proportion of lost parenchyma by 1% leads to a 1.13-time increase in the chance of a significant decrease in sGFR. That is, an increase in this indicator by 10% increases the likelihood of a decrease in the GFR of the affected kidney by 10 mL/min by 3.39 times, and an increase in parenchyma loss by 20% increases the likelihood of a decrease in the sGFR of the affected kidney by 10 mL/min by 11.52 times (Table 3).

**Table 3:** Multivariate logistic regression.

Parameter	LR coefficient	Standard error	*p*-value	OR	2.5% limit of 95% CI	97.5% limit of 95% CI
Sex (m)	0.668	0.639	0.296	1.95	0.568	7.181
Age	0.067	0.038	0.076	1.069	0.996	1.157
BMI	0.069	0.091	0.449	1.071	0.902	1.295
Lost parenchyma, %	0.125	0.03	0	1.133	1.075	1.211
Compression of the PCS	0.128	0.702	0.856	1.136	0.277	4.559

**Figure 3:**
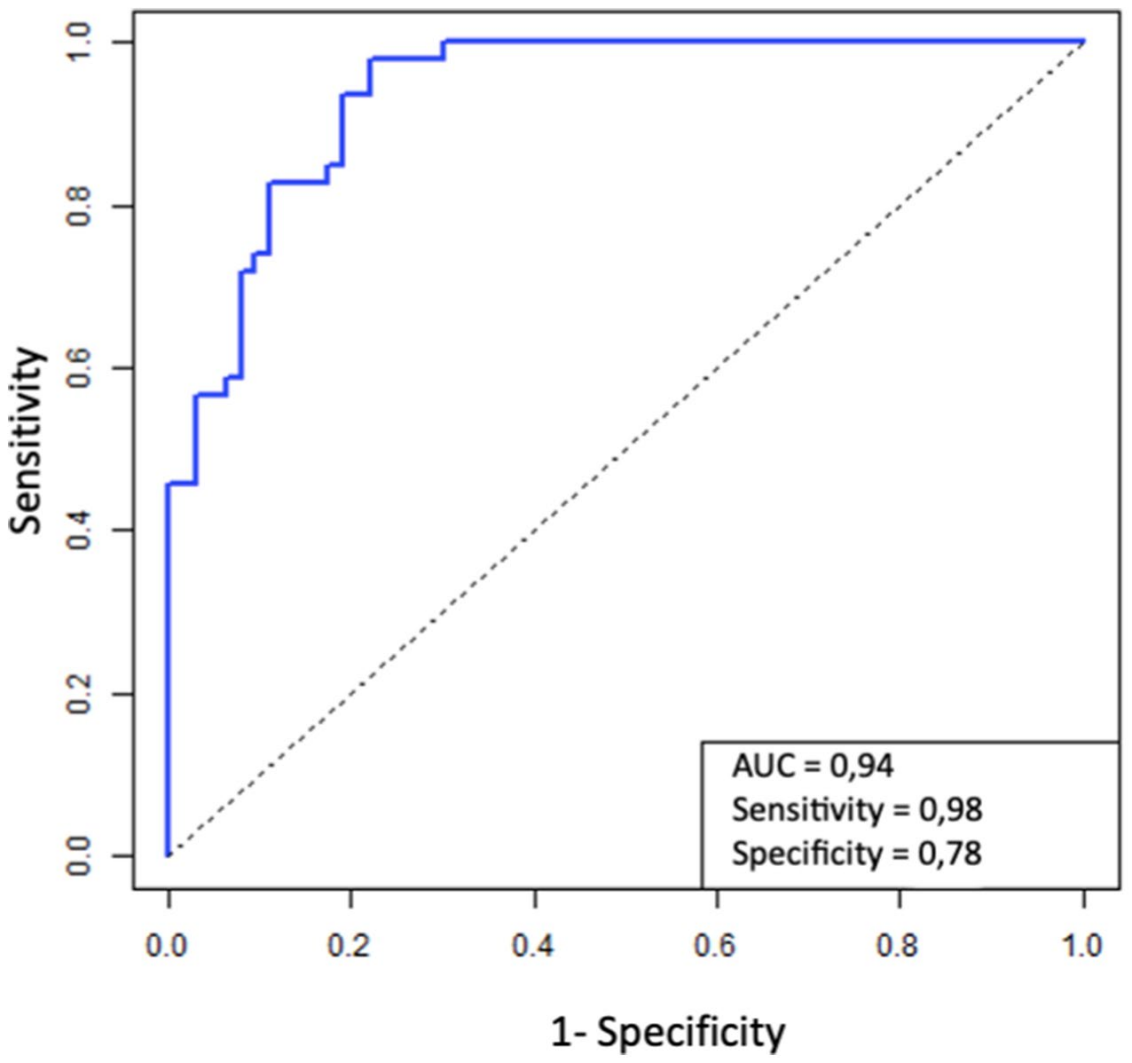
ROC models of the probability of a significant decline in sGFR.

The quality of the selected model parameters was analyzed by calculating AUC (area under the curve) for ROC. The figure shows the ROC for the probability of successful treatment model. The AUC for this ROC is 0.94, which indicates the excellent quality of the model (Figure 3).

## Discussion

Kidney cysts are a common disease but, as of today, there are no clinical recommendations and clear indications for surgical treatment. A decrease in kidney function as a result of compression and atrophy of the renal parenchyma was shown in the experimental work of Gomez et al. The results of a pathological examination of kidney samples with cysts showed signs of atrophy of the renal parenchyma with a significant decrease in the number of nephrons in the zone of compression of the kidney parenchyma by the cyst (21). Al Said et al. in a study involving 561 patients with kidney cysts, noted a decrease in kidney function even in the presence of single cysts. In most studies on this topic, the relationship between kidney function decline and cyst size has been studied (19). In example, Kwon et al. demonstrated decreased renal function in 31 (60.2%) of 50 patients with a mean cyst size of 7.2 cm (18). Chen et al. (16) noted that cysts larger than 2.2 cm were associated with risks of more rapid decline in renal function in a large cohort study reporting 4,274 patients with renal cysts followed over 5 years. Based on the concept of the relative symmetry of the kidney function of a healthy person, according to which each kidney has the same sGFR, the results of our study confirmed the previously obtained worldwide data, showing that the growth of a kidney cyst is accompanied by a decrease in the sGFR of the affected kidney, along with this, our study showed that growth of the cysts leads to atrophy and loss of the renal parenchyma, which is the most likely mechanism for the decline in kidney function. This is indirectly consistent with the results of Wu et al., who showed that a decrease in renal function was observed 10 times more often in the group of patients with progressively growing cysts compared to the group of patients with “stable” cysts (23.3% vs. 2.4%) (13). On average, in the sample of patients presented in our study, cyst growth led to the loss of 49 mL (28%) of the kidney parenchyma, which led to a decrease in sGFR by 11 mL/min compared to a healthy kidney. Statistical analysis also revealed a direct relationship between the maximum cyst size, the volume of lost parenchyma, and the sGFR of the affected kidney. Thus, according to the data of our study, the growth of a kidney cyst can lead to a decrease in the sGFR of the affected kidney, which can be considered as a relative indication for surgical treatment of a simple kidney cyst. For this reason, patients with a simple kidney cyst, based on the obtained results, should be recommended to undergo Tc-99 m (DTPA) renal dynamic imaging in order to assess the decrease in the function of the affected kidney and to determine the indications for surgical treatment of the kidney cyst. As an indication for dynamic nephroscintigraphy, we recommend using a percentage of lost parenchyma of 20% or more (calculated using the segmentation method), since the loss of one-fifth of the volume of the renal parenchyma increases the likelihood of a significant decrease in sGFR by more than 10 times. The obtained data raises the question of the advisability of performing Tc-99 m (DTPA) renal dynamic imaging in order to assess the decrease in the function of the affected kidney, to determine the indications for surgical treatment of a kidney cyst. According to the obtained results, the loss of 20% of the renal parenchyma can be considered as an indication for Tc-99 m (DTPA) renal dynamic imaging.

One limitation of our study is the reduced median total GFR of the study subjects, which may be associated with other causes of impaired renal function, even though the strict exclusion criteria of the study should have excluded patients with the most common causes of reduced renal function. Since cases of multiple renal cysts and their bilateral location are not uncommon in clinical practice, another limitation may be the inclusion criteria for the study, which included exclusively patients with a solitary kidney cyst and a unilateral lesion. Patients referred from the outpatient centers come to our hospital, most often in order to resolve the issue of surgical treatment of a kidney cyst, this circumstance explains the inclusion in the study of mainly patients with large kidney cysts. There is evidence that the volume of the right and left kidneys in healthy people can differ by an average of 5 mL (22), despite such a slight difference, such anatomical asymmetry can lead to a difference in sGFR of the right and left kidneys of 1–2 mL/min, which was also not taken into account in our study. Importantly, a proven decrease in kidney function caused by a cyst cannot be directly considered as an indication for surgical treatment (23). It should be formulated on the basis of further research aimed at answering the question of the effectiveness of surgical treatment to stop the decline in renal function or contribute to its restoration.

## Conclusion

The results show that increase in kidney cyst size causes atrophy of the renal parenchyma and a decrease in sGFR of the affected kidney. An expansion in the volume of the atrophied parenchyma contributes to a decline in the sGFR of the affected kidney. The obtained data raises the question of the advisability of performing Tc-99 m (DTPA) renal dynamic imaging in order to assess the decrease in the function of the affected kidney and to determine the indications for surgical treatment of a kidney cyst. According to the obtained results, the loss of 20% of the renal parenchyma can be considered as an indication for Tc-99 m (DTPA) renal dynamic imaging.

## Data Availability

The original contributions presented in the study are included in the article/supplementary material, further inquiries can be directed to the corresponding author.
